# Effectiveness of Vitamin-E-Doped Polyethylene in Joint Replacement: A Literature Review

**DOI:** 10.3390/jfb6030889

**Published:** 2015-09-08

**Authors:** Antonio Gigante, Carlo Bottegoni, Vincenza Ragone, Lorenzo Banci

**Affiliations:** 1 Clinical Orthopaedics, Department of Clinical and Molecular Science, School of Medicine, Università Politecnica delle Marche, via Tronto 10/A, 60126 Ancona, Italy; E-Mails: a.gigante@univpm.it (A.G.); bottegonicarlo@gmail.com (C.B.); 2 Research and Development Department, Permedica S.p.A., via Como 38, 23807 Merate (LC), Italy; E-Mail: lorenzo.banci@permedica.it

**Keywords:** α-tocopherol, polyethylene, vitamin E stabilized UHMPWE, total joints replacement

## Abstract

Since polyethylene is one of the most frequently used biomaterials, such as in bearing components in joint arthroplasty, strong efforts have been made to improve the design and material properties over the last decades. Antioxidants, such as vitamin-E, seem to be a promising alternative to further increase durability and reduce polyethylene wear and degradation in the long-term. Nevertheless, even if several promising *in vitro* results are available, there is yet no clinical evidence that vitamin-E polyethylenes show these advantages *in vivo*. The aim of this paper was to provide a comprehensive overview on the current knowledge regarding the biological and mechanical proprieties of this biomaterial, underlying the *in vitro* and *in vivo* evidence for effectiveness of vitamin-E-doped polyethylene in joint arthroplasty.

## 1. Introduction

Ultra-high molecular weight polyethylene (UHMWPE) is the most frequently used biomaterial in bearing components in joint arthroplasty.

As with any engineering material, UHMWPE is vulnerable to balances between different properties, most notably oxidation resistance, mechanical toughness, and wear resistance.

Radiation crosslinking of UHMWPE, accompanied by thermal treatments to improve oxidative stability, has decreased wear and the incidence of osteolysis (first-generation cross-linked UHMWPE). One promising approach in improving oxidation resistance is the addition of antioxidants as α-tocopherol (or vitamin E) to the material, thereby preventing oxidation of the polymer while allowing for cross-linking or sterilizing irradiation in the absence of a post-irradiation thermal stabilization (second-generation cross-linked UHMWPE) [1,2].

Vitamin E (VE) is an effective biological antioxidant, helping to prevent the oxidative degradation of cell membrane phospholipids. When added to UHMWPE, VE performs a similar role, helping to prevent oxidation of the polyethylene chains [3,4,5].

There are two methods of incorporating VE into UHMWPE. One is to blend VE with UHMWPE powder before consolidation. Once consolidated, the blend can be irradiated for sterilization or cross-linking [6]. An alternative approach is the diffusion of VE into UHMWPE after radiation cross-linking. Firstly, UHMWPE is irradiated for cross-linking, diffused with VE, then machined into its final form and gamma sterilized [7].

In the last decade an increasing number of commercial brands of highly cross-linked UHMWPE added with VE rose up for hip and knee bearings (Table 1).

The aim of this paper is to provide a comprehensive overview on the current knowledge regarding mechanical and biological advantages of UHMWPE added with VE, underlining the potentiality of this biomaterial in joint arthroplasty.

**Table 1:** Main commercial brands of UHMWPE added with VE for hip and knee bearings. HXLPE: highly cross-linked UHMWPE; XLPE: Cross-linked UHMWPE.

Brand	Raw Material	Incorporated Method	Cross-Linking Grade	Radiation Type	Sterilization Methods
E1™ (Biomet)	GUR 1020/1050	Infused	HXLPE (100 kGy	Gamma-Beam	Gamma-Beam
Vitamys^®^ (Mathys)	GUR 1020	Blended	HXLPE (100 kGy)	Gamma-Beam	Gas plasma
Vivacit-E^®^ (Zimmer)	GUR 1020	Blended	HXLPE (100 kGy)	E-Beam	Eto
Vitelene^®^ (Aesculap)	GUR 1020	Blended	HXLPE (80 kGy)	E-Beam	Eto
Vital-XE^®^ (Permedica)	GUR 1020	Blended	XLPE (60 kGy)/UHMWPE	E-Beam	Eto
ECiMa™ (Corin)	GUR 1020	Blended	HXLPE (120 kGy)	Cold Gamma-Beam	Eto
E-MAX™ (Renovis)	GUR 1020	Blended	HXLPE (100 kGy)	Gamma-Beam	Eto

## 2. Methods

A Medline search was performed on English-language papers published in the last 15 years. Criteria for including studies are reported in Table 2. In the case of a combined keyword, the conjunction AND was inserted. Articles were excluded if they did not meet the inclusion criteria, if they were published prior to January 2000, if they were not written in the English language, and if they were repeated publications from the same group of authors. Study protocol articles, abstract-only publications, and chapters from books were not included. Articles focused on microstructural characterization of materials only, antioxidants other than vitamin E, or alternative formulations of polyethylene were also excluded.

The literature search yielded 424 citations. After screening and reviewing of abstracts, 44 relevant studies were selected for full-text review. Of them only seven were *in vivo* studies (three clinical trials and four *in vivo* animal studies). These articles represent the extent of published evidence on this topic.

**Table 2:** Criteria for including studies in the literature review.

Search Terms	Relevance Field	Language	Publication Dates
Vitamin-E polyethylene, vitamin-E UHMWPE, alpha tocopherol, α-tocopherol	Joint replacement, hip arthroplasty, knee arthroplasty, bearing materials in orthopaedics, tribology	English	February 2015 to January 2000

## 3. Results

### 3.1. Oxidative Stability

The main purpose of using VE in UHMWPE is to prevent oxidative degradation [8,9].

Several types of accelerated-aging *in vitro* studies have shown that, at elevated temperatures and in air-pressure O_2_ or in aqueous hydrogen peroxide solution, VE improve the oxidative resistance of irradiated UHMWPE even in the presence of a small amount of the antioxidant agent [6,10,11,12,13,14,15,16,17,18,19,20,21].

A long-term oxidative stability also has been suggested for this type of polyethylene. Only a minimal VE elution was observed from irradiated, VE-diffused, and terminally gamma-sterilized UHMWPE [21]. Furthermore radiation cross-linking was found to graft VE on UHMWPE (blended with 1% w/w VE), decreasing the possibility of elution and increasing the long-term oxidative stability [22].

An *in vitro* study examined oxidation levels of stabilized UHMWPE prepared by pre-irradiation blending (blended with 0.02, 0.05 and 0.1% w/w VE, and γ-irradiated with 150 kGy/200 kGy ) or post-irradiation diffusion (γ-irradiated with 150 kGy/200 kGy, doped with VE, and annealed at 120 °C) after accelerated aging (70 °C for 2 weeks in presence of pressurized O_2_) and real time aging (in water at 40 °C for 36 months) [23]. Although accelerated aging revealed little to no oxidation in any of stabilized specimens (oxidative index < 0.1), real-time aging protocols showed that VE blends containing 0.02 wt % VE were susceptible to oxidation at 36 months, emphasizing that the long-term oxidative resistance could be affected by levels of VE concentration, as well as by cross-linking dose.

An *in vivo* study showed no oxidation of stabilized-UHMWPE films (blended with 0.8% w/w VE) subcutaneously implanted into rats for six months, confirming the VE ability to prevent oxidation degradation [24].

### 3.2. Mechanical Properties

Wear performance of stabilized UHMWPE has been extensively investigated in several *in vitro* studies (Table 3). UHMWPE added with VE exhibited lower wear rate compared to conventional UHMWPE with no changes in its tribological proprieties after artificial aging [6,13,14,15,16,17,25,26,27,28,29].

**Table 3:** Overview of *in vitro* studies reporting wear rates of different type of UHMWPE.

Authors	Methods	Type of PE	Wear
Oral *et al.* (2006) [26]	Hip simulator, Ø 28 and 36 CoCrMo	(a) Std-PE: γ-ster (b) VE-XPE_ann_: γ-irr 85kGy, infused VE, anneal, γ-ster	(a) 9.54 (clean) and 20.55 (third-body) mg per mc (Ø 28); (b) 0.78 (clean), 5.7 (third-body) mg per mc (Ø 28); 0.97 (clean), 5.1(third-body) mg per mc (Ø 36)
Grupp *et al.* (2014) [13]	Hip simulator, 5 million cycles, Ø 36 CoCrMo & Aluminia (Al)	(a) Std-PE: γ-ster 30kGy (b) XPE_rem_: γ-irr 75kGy, remelt, Eto-ster (c) VE-XPE: blended 0.1% VE, β-irr 80 kGy, Eto-ster	(a) 19 (unaged) and 365.8 (aged) mg per mc (Al) (b) 2 (unaged) and 52 (aged) mg per mc for (Al) (c) 2.5 (unaged) and 2.3 (aged) mg per mc (Al) (b) 3.5 (clean) and 35.8 (third-body) mg per mc (CoCrMo) (c) 3.4 (clean) and 23.5 (third-body) mg per mc (CoCrMo)
Affatato *et al.* (2011) [25]	Hip simulator, 5 million cycles, Ø 28 CoCrMo	(a) Std-PE: Eto-ster (b) XPE_rem_: β-irr 70 kGy, remelt, Eto-ster (c) VE-XPE_rem_: blended 0.1% VE, β-irr 70 kGy, remelt, Eto-ster	(a) 57.7 mg per mc (b) 6.53 mg per mc (c) 22.58 mg per mc
Vaidya *et al.* (2010) [29]	Knee simulator	(a) Std-PE (b) VE-XPE: blended VE	(a) 4.4 mg per mc (unaged) (b) 1.9 mg per mc (aged)
Teramura *et al.* (2007) [27]	Knee simulator	(a) Std-PE (b) VE-PE: blended 0.3% VE	(a) 13 mm^3^ per mc (b) 6 mm^3^ per mc
Micheli *et al.* (2012) [15]	Knee simulator, CR design	(a) Std-PE: γ-ster 25–40 kGy (b) VE-XPE_ann_: γ-irr 100 kGy, infused, VE anneal, γ-ster 30–35 kGy	(a) 26.9 (unaged), 40.8 (aged) mg per mc (b) 2.4 (unaged), 2.5 (aged) mg per mc
Haider *et al.* (2012) [14]	Knee simulator,5 million cycles, TKA CR and PS designs	(a) Std-PE: γ-ster 32 kGy (b) VE-XPE_rem_: γ-irr 100 kGy, infused VE, remelt, γ-ster 25–40 kGy	(a) 19.8 (PS), 39.7 (CR large), 11.3 (CR small) mg per mc (b) 2.7 (PS), 5.98 (CR large), 3.06 (CR small) mg per mc
Schwiesau *et al*. (2014) [28]	Knee simulator, CR design	(a) VE-PE: blended 0.1% VE, γ-ster 30 kGy	(a) 5.6 mg per mc (aged)
Wannomae *et al.* (2010) [16]	Test specimens, Unidirectional pin-on-disk	(a) Std-PE: γ-ster 25-40kGy (b) VE-XPE_ann_: β-irr 100 kGy, infused VE (1.1 wt%), anneal, γ-ster 25–40 kGy	(a) 1.8 (unaged), 41.8 (aged) mg per mc (b) no detectable for either unaged and aged conditions
Oral *et al.* (2004) [17]	Test specimens, Bidirectional pin-on-disk	(a) VE-XPE: γ-irr 65 kGy, infused VE, γ-ster 27 kGy (b) VE-XPE: γ-irr 100 kGy, infused VE, γ-ster 27 kGy	(a) 1.7 (unaged), 1.9 (aged) mg per mc (b) about 0.9 mg per mc (aged and unaged)
Oral *et al.* (2005) [6]	Test specimens, Bidirectional pin-on-disk	(a) VE-XPE: blended 0.1 and 0.3% VE, γ-irr 100 kGy	(a) 2.1 (0.1% VE) and 5 (0.3% VE) mg per mc (aged)

VE: vitamin E; Std-PE: conventional ultra-high molecular weight polyethylene (UHMWPE) (not receiving any irradiation dose irrespective of the sterilization method); XPE: cross-linked UHMWPE; VE-PE: PE added with VE; VE-XPE: cross-linked UHMWPE added with VE. CR: Cruciate retaining; PS: posterior-stabilized.

In an hip simulator study, higher wear for VE blended UHMWPE (0.1 wt %), compared to cross-linked and remelted UHMWPE, was observed (22.58 *vs* 6.53 mg per mc, *p* < 0.002) (Table 3) [25]. However, a most recent study did not show any advantages for irradiated and re-melted UHMWPE, which showed a dramatical increased in wear after accelerated aging compared to irradiated VE-blended UHMWPE (0.1 wt %) (2.3 *versus* 52 mg per mc, *p* < 0.05) (Table 3) [13].

On the other hand, a higher dose of antioxidant (0.3 wt %) was found to reduce the wear resistance of irradiated blends compared to a lower dose of VE (0.1 wt %) (5 *versus* 2.1 mg per mc, *p*-value = 0.018) (Table 3) [6].

Although conventional UHMWPE has showed high tensile and fatigue strength in its unaged form, its mechanical properties were found deteriorated after accelerated aging [6,10,14,15,17,18,19,26,30]. Ultimate tensile strength (UTS) values of conventional UHMWPE in its unaged state ranged from 50.2 to 52MPa and from 33 to 34.7 MPa after accelerated aging [15,26]. Stress intensity factor at fatigue crack inception (Δ*k*_i_) decreased from 1.09–1.29 MPa∙m^1/2^ to 0.18–0.53 MPa∙m^1/2^ after artificial aging [15,17]. otherwise, no significant changes in mechanical and fatigue strength were seen for irradiated and VE-stabilized UHMWPE after accelerated aging (UTS: 43.4–46 MPa *versus* 45 MPa; Δ*k*_i_: 0.65–0.77 MPa·m^1/2^ *versus* 0.61–0.87 MPa·m^1/2^) [15,17,26]. The incorporation of VE didn’t change the mechanical properties of irradiated UHMWPE, supporting the lack of oxidative degradation of those properties.

Additionally, since the loss of crystallinity during melting was avoided, irradiated and VE-stabilized UHMWPE has shown higher mechanical properties compared to irradiated and remelted UHMWPE. In fact, a significantly lower fatigue resistance was found for 100 kGy irradiated and remelted UHMWPE (Δ*k*_i_: 0.56 MPa∙m^1/2^) [17,26].

However a higher plasticity was observed for blended UHMWPE at VE concentrations equal to or above 0.3 wt %. Conversely, to virgin UHMWPE that has shown to have a dose-dependent decrease in elongation (%) and work-to-failure (kJ/m^2^), mechanical proprieties of 1.0 wt % VE blended UHMWPE increased until to 100 kGy and then decrease to comparable values of un-irradiated 1.0 wt % blended UHMWPE at 150 kGy [8,31].

### 3.3. Biocontamination

One of the reasons for failure in joint replacement is septic loosening of the prosthetic implant. Deep infection is caused by the bacterial adhesion onto the prosthetic surface. Following adhesion, bacteria can grow and produce a structured community enclosed in a stable matrix, called biofilm, a survival mechanism for microorganisms on biomaterials surfaces.

Thus, reducing the bacterial adhesive ability, the risk of periprosthetic deep infection is reduced. Data on microbial adhesion on UHMWPE added with VE are still conflicting.

Some authors have shown VE blended UHMWPE (not cross-linked UHMWPE, blended with 0.1% and 0.5% w/w VE) decreased *in vitro* the adherence ability of some of the most common bacteria involved in periprosthetic joint infections, as *Staphylococcus epidermidis, Staphylococcus aureus* and *Escherichia coli*, in comparison with virgin UHMWPE [32,33,34]. In addition, a decreased adhesion on VE-blended UHMWPE has been also found for *Candida albicans* [34].

These results highlight that VE, other than to minimize UHMWPE oxidation, can change the surface of the substratum and microbial adhesion process, limiting the extent of subsequent infection.

Other authors have found that VE affected the adherence of *Staphylococcus epidermidis* and *Staphylococcus aureus* on UHMWPE in a variable way in the different species and strains. Gomez-Barrena *et al.* [35,36] reported a reduction in bacterial adherence to both VE-infused (3 and 0.4% w/w) and blended UHMWPE (0.1% w/w) independently of the concentration in use, but the results showed important intra-species differences.

Lastly, under physiologically relevant conditions, VE-blended highly cross-linked UHMWPE (γ irradiation dose: 150 kGy, 75 kGy) didn’t prevent or reduce the attachment or formation of bacterial biofilms of a clinically-relevant strain of methicillin-resistant *Staphylococcus aureus* compared to virgin polymers (conventional UHMWPE, highly cross-linked UHMWPE with γ-rays at 75 kGy/150 kGy, polyetheretherketone) [37]. Although these results suggest that the addition of VE to UHMWPE may not to prevent implant-related infection, further investigations are required to draw conclusions about the ability of VE to reduce risk infection.

### 3.4. Biocompatibility

Alpha-tocopherol (VE) is a natural antioxidant fundamental for human metabolism. Added to the polymer VE may, however, undergo different chemical transformations during manufacturing and sterilization of final medical devices than in human metabolism.

One concern regarding VE-stabilized UHMWPE involves its possible *in vivo* elution from the polymer and their local and systemic effects.

#### 3.4.1. *In Vitro* Studies

Wolf *et al.* [38], firstly, in 2002, evaluated the biocompatibility behavior of VE-stabilized UHMWPE samples prepared under the same conditions as applied for the production and sterilization of hip-cups (blended with 0.8% w/w VE, sterilized with γ-rays). The stabilization of UHMWPE with VE did not influence the proliferation and mitochondrial activity of mouse fibroblasts, as well as the membrane integrity of cells seeded on VE-stabilized UHMWPE specimens, compared to non-toxic material (negative control). The genotoxic activity was also not affected. In contrast, cell adhesion and cell spreading were diminished, suggesting that this material is biocompatible but not bioactive.

Later, the same authors performed a similar experiment using two different lines of human fibroblasts, proving once again no cytotoxicity for VE-stabilized UHMWPE compared to virgin UHMWPE [39].

Conversely, Gazzano *et al.* [40] failed to prove any advantages of VE supplementation. A significant toxic effect was seen for all investigated types of UHMWPE (conventional UHMWPE, oxidized UHMWPE, highly cross-linked UHMWPE with β-rays at 75 kGy, blended with 0.5% w/w VE). Slides of standard UHMWPE, or its derivate, (1 cm^2^) evoked an oxidative stress in human osteoblasts cells incubated for 72 h in the presence of these materials, compared to non-stimulated control cells.

A recent study has proven that both liquid VE (at non-cytotoxic dose of 800 µM) and VE-stabilized UHMWPE (blended with 0.1%, 0.3%, 3% w/w VE) were able to reduce osteolytic cytokines release from human peripheral blood mononuclear cells [41]. The production of inflammatory cytokines including tumor necrosis factor-alpha (TNF-α), interleukin 1β (IL-β), IL-6 and IL-8 was significantly reduced in cells stimulated with wear debris of VE-stabilized UHMWPE compared to virgin UHMWPE particles. This trend was also observed when VE was added as a liquid to UHMWPE wear particle-stimulated mononuclear cells.

The potential advantages of the use of VE as additive for UHMWPE were also investigated by Renò *et al.* [42] that studied the activation of granulocytes obtained from human peripheral venous blood cultured in the presence of bulk samples of normal UHMWPE and VE-stabilized UHMWPE samples (blended with 0.5% w/w VE, un-irradiated). VE-stabilized UHMWPE was able to increase the inflammatory protease MMP-9, compared to virgin UHMWPE. These results suggest that VE could modulate the *in situ* tissue bone remodeling and immunity response through MMP-9 release [43,44].

Later, the same authors found that VE-stabilized UHMWPE (blended with 0.1% w/w VE) adsorbed slightly less immunoglobulin G (IgG), than virgin UHMWPE [45]. Since human macrophages’long-term adhesion is mediated by IgG, VE-stabilized UHMWPE surfaces can be less prone to foreign-body reaction compared to virgin UHMWPE.

#### 3.4.2. *In Vivo* Studies

Wolf *et al.* [24] first investigated the *in vivo* biocompatibility of stabilized UHMWPE implants. Stabilized (blended with 0.8% w/w VE), as well as un-stabilized, UHMWPE films were implanted subcutaneously into rats after sterilization with γ-rays at 25 kGy. The implants were well-tolerated and definitely encapsulated two weeks after surgery. No difference in morphology and reactivity of surrounding connective tissue was observed between the two types of implants, showing no evidence for a toxic behavior of VE-stabilized UHMWPE. The elution of VE from the implant was found low enough to ensure an adequate lifetime stabilization and did not induce *in vivo* adverse effects.

Later, Jarrett *et al.* [46] demonstrated that direct injection of solubilized VE into rabbit knees did not alter the histological appearances of surrounding soft tissue. In a second experiment, no difference was found in tissue response to virgin UHMWPE plugs or VE-diffused highly cross-linked UHMWPE (β-irradiated to 100 kGy and homogenized at 120 °C) plugs implanted subcutaneously in rabbits after sterilization with γ-rays (25 to 40 kGy).

Finally, using a canine total hip replacement model, no difference in bone ingrowth of cups was seen between re-melted highly cross-linked UHMWPE liners (β-irradiated to 100 kGy and sterilized by γ irradiation) and VE-diffused highly cross-linked UHMWPE liners (VE concentrations: 1.4 and 0.7% w/w) after three months of implantation time.

These results suggested that topical administration of VE, as well as VE elution from highly cross-linked UHMWPE, did not affect the local tissue response or ingrowth of the surrounding bone into the porous prosthesis components.

Bichara *et al.* [47] first investigated *in vivo* osteolysis caused by participles debris from gamma-sterilized highly cross-linked VE-stabilized UHMWPE (approximate 0.8% by weight, diffused after 100 kGy irradiation). Using a murine calvarial bone model they found an inferior bone resorption area compared to participles debris from virgin highly cross-linked UHMWPE (β-irradiated to 100 kGy and melt-stabilized at 150°C). Histological analysis revealed a significant greater amount of inflammatory fibrous tissue overlaying calvaria for virgin UHMWPE wear particles.

Using the same animal model a more recent study [48] investigated the biological response to participles debris of three commercially available UHMWPE: conventional UHMWPE, highly cross-linked UHMWPE (70 kGy) and VE-stabilized highly cross-linked UHMWPE (blended with 0.5% w/w, irradiated with a dose of 70 kGy). The same mass of wear debris (1 mg) was injected into the exposed calvaria area of animals. According to the previous study, a greater risk of particle-induced osteolysis and heightened inflammatory response was observed in the highly cross-linked UHMWPE group. The VE-stabilized highly cross-linked UHMWPE group showed no significant difference in inflammatory and osteolytic responses when compared to the conventional UHMWPE group indicating that the possible elution of VE *in vivo* from wear processes did not induce adverse effects.

### 3.5. Clinical Evidence

Most recently, clinical trials have been conducted to investigate the wear behavior of the first commercialized antioxidant-stabilized cross-linked UHMWPE liner (E-Poly™ and later E1™ with some changes in manufacturing, Biomet^®^) using the radiostereometry analysis (RSA).

Lindalen *et al.* [49] conducted a randomized study to compare the head penetration inside VE-diffused UHMWPE liners between 2 different head size. 50 patients were randomized to receive either a 32 or 36 mm head (Biolox^®^ Delta ceramic head) articulating with E-Poly liners (β-irradiated and VE-diffused UHMWPE). After a period of bedding-in, no significant difference in wear was found until 24-months follow-up, suggesting that the most of the wear measured is the effect of bedding that occurs during the first three months after surgery. Although a small, significant difference in total 3D direction was seen between 32 mm and 36 mm heads (0.195 mm *versus* 0.158 mm, *p* = 0.045) at two-year follow-up, this difference was considered not clinically relevant.

Later, Sillesen *et al.* [50] conducted a prospective multicenter study in order to evaluate the wear of VE-diffused UHMWPE liners (E1™, Biomet, Table 1) in THA. Patients received either a 32 mm cobalt-chromium head or a 32 mm ceramic head. There was no difference in femoral head penetration between cobalt-chrome and ceramic head at three-year follow-up (median: −0.0280 mm *versus* −0.043 mm; *p* = 0.450) indicating a minimal VE-diffused UHMWPE liners wear.

Only a RCT has been conducted [51] to compare the wear properties of VE-diffused UHMWPE liner (E1™, Biomet, Table 1) with those of highly cross-linked UHMWPE liner (Marathon™, Depuy) articulating with the same 32 mm cobalt-chrome head. Femoral head penetration in the medial/lateral and vertical directions was significantly lower for VE-diffused UHMWPE liners up to two-year follow-up. Mean head penetration in medial/lateral direction for VE and control group were 0.05 mm and 0.15 mm, respectively (*p* = 0.004). In the vertical direction these were 0.01 mm and 0.09 mm, respectively (*p* = 0.035). However, the difference in total head penetration calculated from the three axes did not reach statistical significance (*p* = 0.09).

## 4. Conclusions

The results of this extensive literature review support the use of VE-stabilized UHMWPE in total joints arthroplasty.

Several *in vitro* studies have shown VE-stabilized UHMWPE had a higher oxidative resistance of that of irradiated UHMWPE, as well as equivalent wear and improved mechanical strength compared to irradiated and melted UHMWPE.

Furthermore *in vitro* and animal studies did not show adverse biological responses to VE-stabilized UHMWPE, of which debris particles also had a lower osteolytic potential.

An additional biological advantage refers to the potential ability of this biomaterial to limit the bacterial adhesion onto prosthetic surfaces, reducing the risk of periprosthetic deep infection.

VE-stabilized UHMWPE is a biocompatible material with good mechanical, wear, and oxidative proprieties. Nevertheless, even if several promising *in vitro* results are available, there is still a paucity of clinical evidence to prove efficacy of UHMWPE added with VE *in vivo*.

To our knowledge no clinical studies proving the clinical performance of this material in total knee arthroplasty has been published yet. Randomized controlled studies in hip artroplasty also are lacking. To our knowledge only one randomized controlled study (level 1) has been performed, showing a lower initial head penetration and lower wear for VE-stabilized UHMWPE compared to virgin material.

Well-designed randomized studies with longer follow-up are required to demonstrate a higher clinical performance of vitamin E polyethylene bearings compared to current highly cross-linked polyethylene components.
